# Supplementary material for: Detecting Phytophthora cinnamomi associated with dieback disease on Carya cathayensis using loop-mediated isothermal amplification
Source: PLoS One. 2021 Nov 16;16(11):e0257785. doi: 10.1371/journal.pone.0257785 (PMC8594852; doi:10.1371/journal.pone.0257785)
Supplement: S1 Table — (DOCX) [file pone.0257785.s001.docx]

Table S1. GenBank accession numbers of taxa used in phylogenetic analyses

| Species name | Strain number | GenBank accession number | |
| --- | --- | --- | --- |
|  |  | ITS | *COI* |
| *P. alni* | CBS 117375 | HQ643143 | HQ708217 |
| *P. cactorum* | CBS 110121 | HQ643168 | HQ708238 |
| *P. capsici* | CBS 111335 | HQ643186 | HQ708254 |
| *P. cinnamomi* | CBS 40348 | HQ643190 | HQ708258 |
| *P. cinnamomi* | **ST402** | **MT675107** | **MT683171** |
| *P. infestans* | CBS 36651 | HQ643247 | HQ708309 |
| *P. melonis* | CBS 58269 | HQ643283 | HQ708336 |
| *P. multivora* | CBS 124094 | FJ237521 | FJ237508 |
| *P. nicotianae* | CBS 101655 | HQ643303 | HQ708354 |
| *P. ramorum* | CBS 101553 | HQ643339 | HQ708387 |
| *P. sojae* | CBS 38261 | HQ643347 | HQ708395 |
| *Pythium aphanidermatum* | CBS 28779 | HQ643439 | HQ708486 |
