# Supplementary figures and images for: Detecting Phytophthora cinnamomi associated with dieback disease on Carya cathayensis using loop-mediated isothermal amplification
Source: PLoS One. 2021 Nov 16;16(11):e0257785. doi: 10.1371/journal.pone.0257785 (PMC8594852; doi:10.1371/journal.pone.0257785)

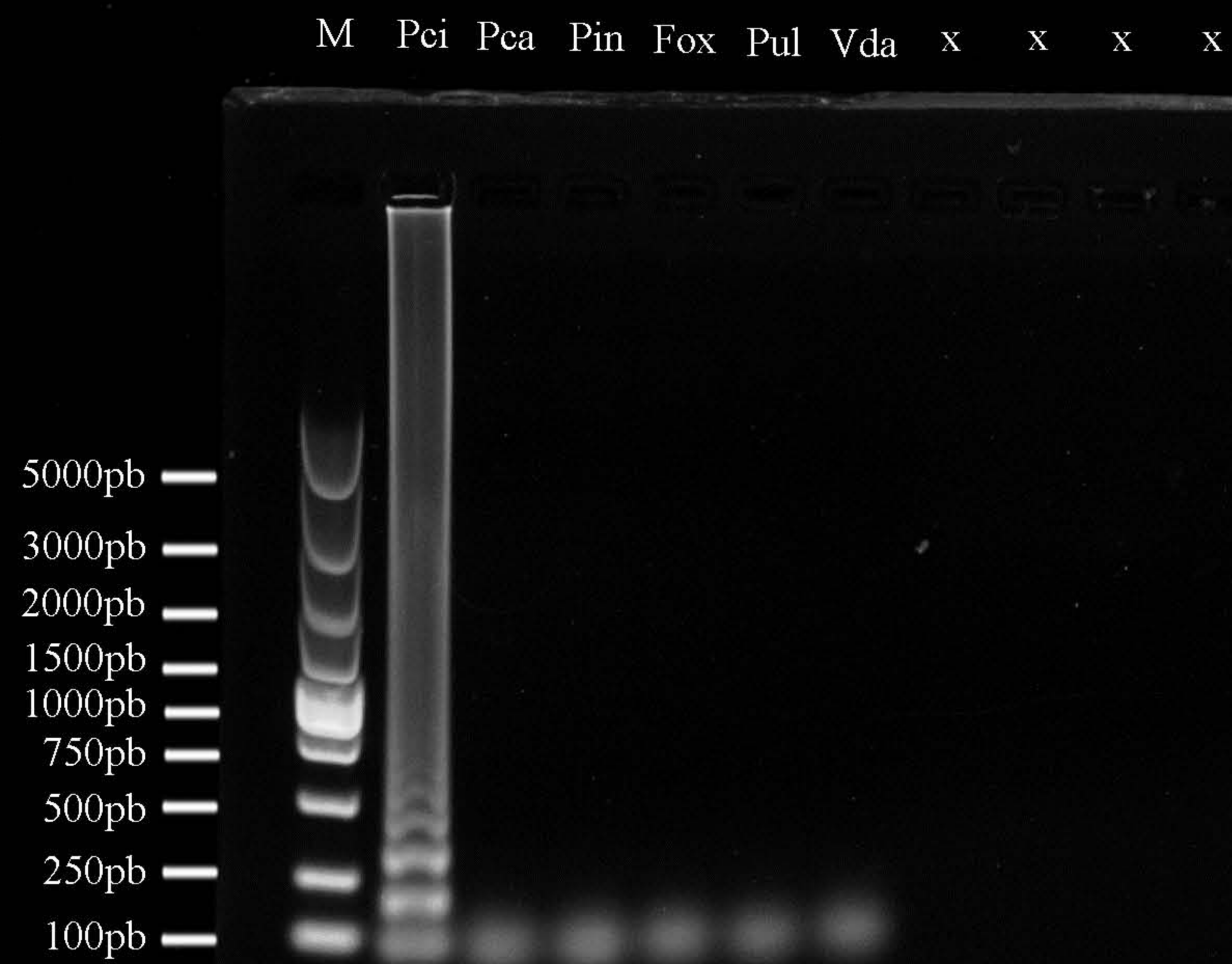

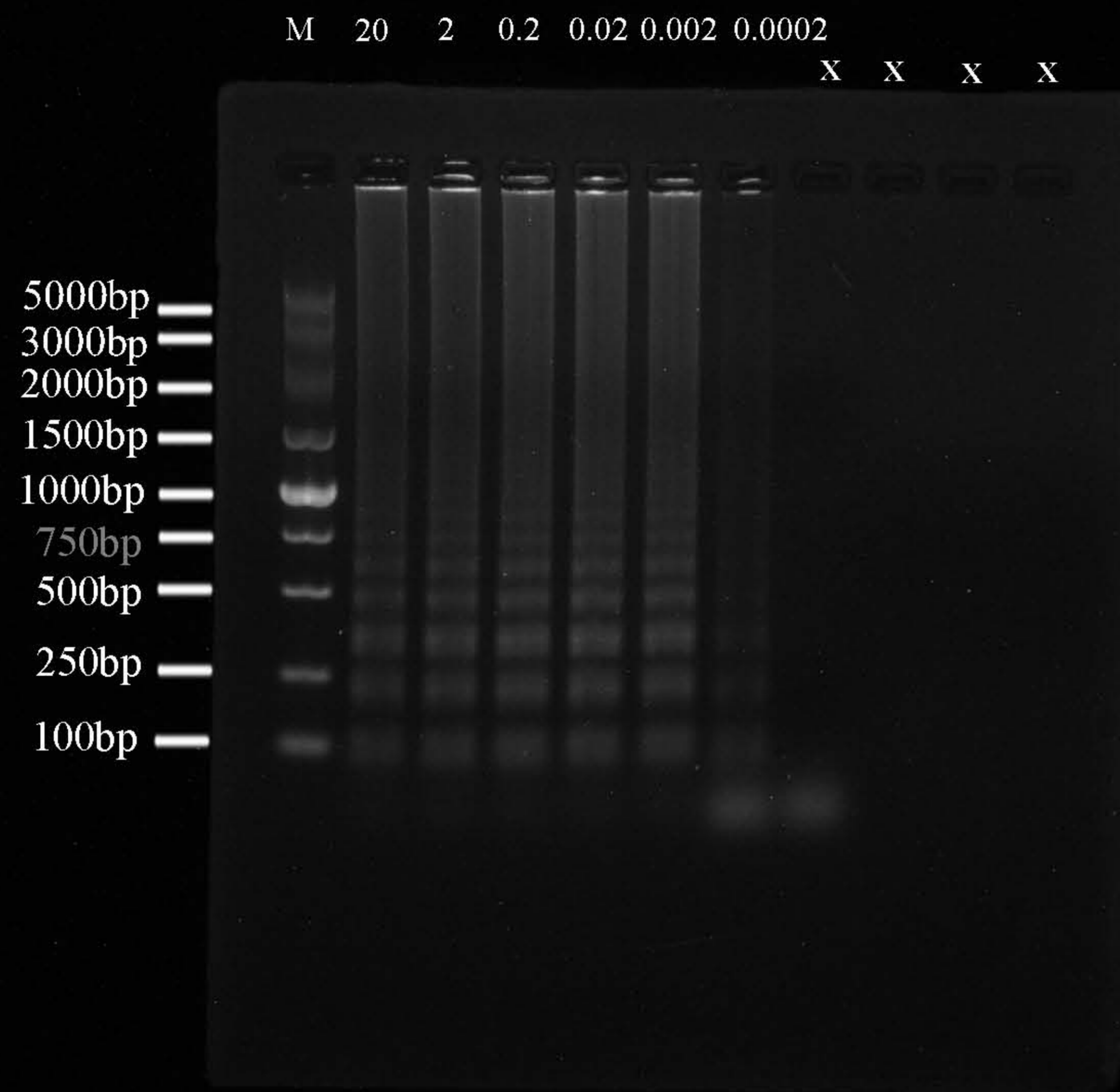

Supplement: S1 Raw images — (PDF) [file pone.0257785.s002.pdf]
